# Porous Ultrahigh Molecular Weight Polyethylene/Functionalized Activated Nanocarbon Composites with Improved Biocompatibility

**DOI:** 10.3390/ma14206065

**Published:** 2021-10-14

**Authors:** Wangxi Fan, Xiuqin Fu, Zefang Li, Junfei Ou, Zhou Yang, Meng Xiang, Zhongli Qin

**Affiliations:** 1 School of Materials Engineering, Jiangsu University of Technology, Changzhou 213001, China; fwx@jsut.edu.cn (W.F.); oujunfei_1982@163.com (J.O.); zhouyang@jsut.edu.cn (Z.Y.); xiangmeng10@just.edu.cn (M.X.); 2 School of Life Science and Technology, Wuhan Bioengineering Institute, Wuhan 430415, China; fu_2021@foxmail.com; 3 School of Computer Engineering, Jiangsu University of Technology, Changzhou 213001, China; zflz0000@foxmail.com; 4 School of Electronics and Information Engineering, Hubei University of Science and Technology, Xianning 437100, China

**Keywords:** porous materials, activated nanocarbon, hydrophilicity, biocompatibility in vitro

## Abstract

Ultrahigh molecular weight polyethylene (UHMWPE) materials have been prevalent joint replacement materials for more than 45 years because of their excellent biocompatibility and wear resistance. In this study, functionalized activated nanocarbon (FANC) was prepared by grafting maleic anhydride polyethylene onto acid-treated activated nanocarbon. A novel porous UHMWPE composite was prepared by incorporating the appropriate amount of FANC and pore-forming agents during the hot-pressing process for medical UHMWPE powder. The experimental results showed that the best prepared porous UHMWPE/FANC exhibited appropriate tensile strength, porosity, and excellent hydrophilicity, with a contact angle of 65.9°. In vitro experiments showed that the porous UHMWPE/FANC had excellent biocompatibility, which is due to its porous structure and hydrophilicity caused by FANC. This study demonstrates the potential viability for our porous UHMWPE/FANC to be used as cartilage replacement material for biomedical applications.

## 1. Introduction

Artificial joints are used to replace the malfunctioning bones, enabling thousands of people to enjoy an active lifestyle [1,2,3,4]. The latest technologies in the field of joint replacement include bone integration of artificial joints, improvements in bone replacement materials, implants, and rehabilitation devices, and joint replacement technology based on dynamic bone synthesis models [5,6]. New bone materials, such as PVA-H [7,8,9] and UHMWPE, with a high modulus of elasticity, biocompatibility, and mechanical properties, were studied systematically and in depth [10,11,12]. Ultra-high molecular weight polyethylene (UHMWPE) is the most common material used for the artificial joint bearing component, with the advantage of unique characteristics and favorable properties, such as biocompatibility, chemical stability, high wear resistance, and low friction [10,11,12,13,14]. However, the low hydrophilicity and mechanics properties of UHMWPE easily lead to artificial joint replacement failure [15,16].

Porous UHMWPE simulating as closely as possible the porous articular cartilage is a promising composite polymer used for various implant materials, which can provide excellent bearing capacity for human joints. Various porous UHMWPE with appreciate porosity have been prepared by non-dense injection molding methods with higher efficiency, template-leaching methods, and non-foaming leaching techniques [17,18,19,20,21,22]. NaCl is one biologically compatible and innocuous pore-forming filler to fabricate the porous structure formation in UHMWPE with thermal compression [15,16,19,21].

Unfortunately, due to the appearance of holes, the integrity of UHMWPE is destroyed, which affects its hydrophilicity and mechanical properties. Therefore, some investigations found that hydroxyapatite [23,24], graphene [15,25], chitosan [26,27] and bioactive glass [28] can significantly improve the Young’s modulus, shear modulus, hardness, friction coefficient, or osteogenic properties of the porous UHMWPE. In contrast to hydroxyapatite, graphene, chitosan, or bioactive glass, as a commercial nanoparticle, activated nanocarbons (ANCs) with many hydrophilic active groups on their surface could be commonly used as the reinforcing filler for polymer [29,30,31]. However, ANCs are hydrophilic with the water-soluble units, and they are difficult to evenly disperse in hydrophobic UHMWPE during the fabrication process. We found that functionalized activated nanocarbons (FANCs) prepared by grafting maleic anhydride polyethylene (PE-g-MAH) onto acid-treated activated nanocarbons (ATANCs) can be evenly in UHMWPE, which can improve the hydrophilicity and mechanical properties of UHMWPE composite [29,30,31].

The main purpose of this study is to fabricate porous UHMWPE materials with excellent hydrophilicity and mechanical properties, NaCl is used as pore-forming agent to endow UHMWPE material with a porous structure, and FANC is used as the reinforcing agent to improve the hydrophilicity and mechanical properties of UHMWPE. Porous UHMWPE/FANC composites were prepared by incorporating the appropriate amount of FANC and pore-forming agents during the hot-pressing process of medical UHMWPE powder. The porosity, morphological, tensile properties, hydrophilicity, and biocompatibility of porous UHMWPE/FANC in vitro were evaluated.

## 2. Materials and Methods

### 2.1. Materials

The medical UHMWPE powder with a molecular weight of 5.0 × 10^6^ was purchased from the Shenzhen Teli New material Technology Co., Ltd. from Shenzhen, China. ANCs with a specific surface area of 500 m^2^/g were supplied by Jiangsu Enkai Equipment Co., Ltd. from Taizhou, China. PE-g-MAH with a grafting rate of 0.5 wt.% was purchased from the Coace Chmesitry Co., Ltd. from Fujian, China. Mouse embryonic fibroblast cells (3T3 cells), fetal bovine serum (FBS), Dulbecco’s modified Eagle’s medium (low glucose, DMEM), and 3-(4, 5-dimethyl-2-thiazolyl)-2, 5-diphenyl-2-H-tetrazolium bromide (MTT) purchased from Aladdin reagent website were produced by GIBCO, from New York, NY, USA.

### 2.2. Sample Preparation

#### 2.2.1. Preparation of FANC

Detailed experimental procedures for preparation of FANC*^x^* were reported in our previous investigations [29,30,31]. The superscript *x* denotes the weight ratio of PE-g-MAH to ATANC used in the preparation processes of FANC*^x^* particles, and the values of *x* were 5, 10, 12.5, 15, and 20. It can be seen from the Fourier-transform infrared spectroscopy (FT-IR) and the morphology on the surfaces by transmission electron microscope (TEM) (Japan Electronics, Tokyo, Japan) results that the prepared FANC*^x^* exhibited excellent structure when *x* = 12.5. Therefore, only FANC^12.5^ was used in the preparation of UHMWPE/FANC*^x^* in subsequent studies.

#### 2.2.2. Preparation of UHMWPE/FANC^12.5^

Varying amounts of FANC^12.5^ and medical UHMWPE powder were dispersed and dissolved in decalin at 135 °C for 90 min, in which 0.1% BHT was added as an antioxidant. The gel was ground to particle sizes fine enough to pass through a 100-mesh shaker by ball-milling after cooling to room temperature. The powder was poured into n-hexane under stirring conditions. A top liquid layer and a solid bottom layer were obtained after filtration. The solid layer was washed with acetone 3 times, and then dried at 65 °C for 8 h to remove the remaining acetone solvent.

#### 2.2.3. Preparation of Porous UHMWPE/FANC^12.5^

NaCl particles were ground fine enough to pass through a 100-mesh shaker. Varying amounts of NaCl particles together with modified UHMWPE were fully mixed and pressed in a model with a ZG-50 precision automatic tablet press (Zhenggong electromechanical Co., Ltd., Dongguan, China). After being pressed for 10 min at 10 MPa and 25 °C, the temperature was gradually raised to 180 °C; then, the pressure and temperature were maintained for 20 min. The specimens were cooled in a drying box for 10 min after the pressure was removed, then fully immersed in distilled water for 96 h, with the water replaced by fresh distilled water every 12 h in order to dissolve and removed NaCl particles as much as possible. The porous UHMWPE/FANC^12.5^ specimens were successfully prepared after being cleaned 3 to 4 times, and dried in a constant temperature incubator at 37 °C.

### 2.3. Testing and Characterization

#### 2.3.1. FT-IR Spectroscopy and Morphological Analysis

Detailed experimental procedures for FT-IR spectroscopic measurements and the morphology on the surfaces by TEM of FANC^x^ were reported in our previous investigations [29,30,31].

#### 2.3.2. Porosity Testing of Porous UHMWPE/FANC^12.5^

A 3H-2000PM1 analyzer (Beishide Instrument, Beijing, China) was used to evaluate the porous texture of the UHMWPE/FANC^12.5^ specimens. Prior to analysis, all samples were degassed in ultrahigh purity nitrogen for 30 min at 90 °C, and then for 90 min at 200 °C. The porous texture of the UHMWPE/FANC^12.5^ specimens was determined with a 5-point Brunauer–Emmett–Teller (BET) measurement with ultrahigh purity nitrogen as the adsorbate and liquid nitrogen as the cryogen at −197 °C [32]. In order to obtain a clear pore morphology of the porous UHMWPE/FANC^12.5^ sheet surface and tensile fractured surface, a TECNAI G20 scanning electron microscope (FEI, Hillsboro, OR, USA) was used.

#### 2.3.3. Mechanical Property Testing

The tensile properties of the porous UHMWPE/FANC^12.5^ specimens, which were made into dog-bone splines of 8 mm × 4.4 mm × 1.4 mm, were measured using a tension testing machine (HT-9112, Hung-Ta, Taibei, China). Five samples of each specimen were measured and averaged during the tensile strength measurements. Subsequently, the stress orientations of the fractured surface were analyzed.

#### 2.3.4. Hydrophilicity Testing

Static water contact angles (CAs) on the sample surface were measured using an automatic contact angle measuring instrument (DSA 20, Kruss, Heidelberg, Germany). Samples of 8 μL of deionized water were used for measurements. The *CA* was measured at five different positions on the surface of the sample, and the average value was used as the reported result [33].

#### 2.3.5. Cell Viability Testing

Cell viability (CV) was tested by colorimetric assays using MTT. The porous UHMWPE/FANC^12.5^ specimens with areas of 1 cm^2^ were placed into each well of a 24-well plate. All specimens were washed 3 times with sterile PBS and were exposed to ultraviolet sterilization for 3–4 h, and then cultured in medium overnight. The 3T3 cells were widely used in the cell activity experiments of porous materials due to their strong contact inhibition and they can clearly identify transformed cells [34,35,36]. The 3T3 cells were cultured on the specimen surface at a density of 1 × 10^5^ cells per well for 1, 3 and 5 days in a 5% CO_2_ incubator at 37 °C. The culture media were replaced with fresh media every 2 days. After treatment, MTT solution (0.5 mL) was added to each well and cultured at 37 °C for 3–4 h. A sample of 1 mL of dimethyl sulfoxide was dropped into each well to dissolve intracellular formazan. Absorption was recorded using a micro plate reader at 570 nm [11]. Cell viability percentage was determined using the equation:

CV = *OD_m_*/*OD_r_* × 100 (1)

where *OD_m_* and *OD_r_* is the optical density of the treated and untreated cells, respectively. Thin slices with 2 mm thickness of porous UHMWPE/FANC^12.5^ and porous UHMWPE were taken for cell viability test.

#### 2.3.6. Fluorescence Imaging

After 1, 3 and 5 days of culture on the porous UHMWPE/FANC^12.5^ specimens, cells were washed with warm PBS to remove non-adherent cells and fixed with 4% paraformaldehyde. After the cells were fixed, live cells were stained with acridine orange and were observed under a fluorescent microscope (Leica, Wetzlar, Germany).

## 3. Results and Discussion

### 3.1. FT-IR Spectroscopy and Morphological Analysis

**Figure 1:**
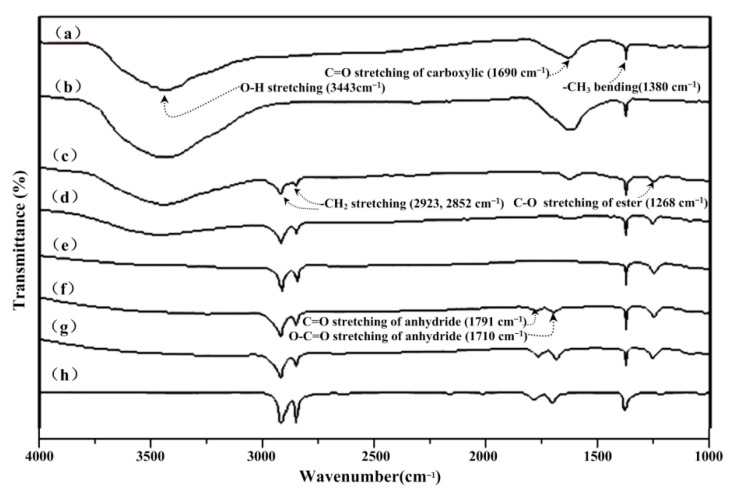
FT-IR spectra of (**a**) ANC, (**b**) ATANC, (**c**) FANC^5^, (**d**) FANC^10^, (**e**) FANC^12.5^, (**f**) FANC^15^, (**g**) FANC^20^, and (**h**) PE-g-MAH specimens.

Figure 1 illustrates typical FT-IR spectra of ANC, ATANC, FANC*^x^*, and PE-g-MAH specimens. Excepting the similar characteristic peaks, three new peaks present at 1268, 2852, and 2923 cm^−1^, respectively, which indicates the successful graft of the ATANC and PE-g-MAH (see Figure 1). The reappearance of C=O and O-C=O stretching bands of maleic anhydride groups (i.e., 1791 and 1710 cm^−1^, respectively) was most likely due to the over-dosage of PE-g-MAH during the functionalization processes of FANC*^x^* specimens [29,30,31].

**Figure 2:**
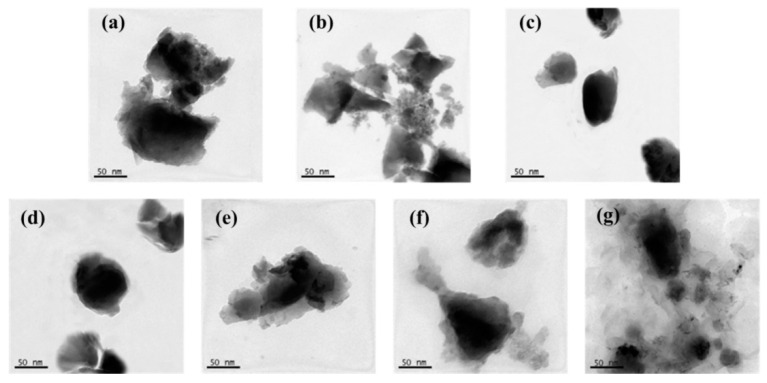
TEM micrographs of (**a**) ANC, (**b**) ATANC, (**c**) FANC^5^, (**d**) FANC^10^, (**e**) FANC^12.5^, (**f**) FANC^15^, and (**g**) FANC^20^ specimens.

Figure 2 exhibits typical TEM micrographs of ANC, ATANC, and FANC^x^ specimens. After being modified by PE-g-MAH, some translucent resins were found attached to the surfaces of ANC particles, wherein the content of attached translucent resins increased gradually as the values of x increased (see Figure 2c–g). Specially, translucent resins were found to fully surround and overwrap the ATANC as x > 12.5 (i.e., the weight ratios of PE-g-MAH to ATANC were greater than 12.5), which is not conducive to the dispersion of FANC^x^ in UHMWPE.

Detailed FT-IR spectra analysis of ANC, ATANC, FANC^x^, and PE-g-MAH and morphological analysis of ANC, ATANC, and FANC^x^ were reported in our previous investigations [29,30,31].

### 3.2. Balance between Porosity and Tensile Properties

Porous permeation is one of the most important characteristics in natural articular cartilage models. A low *P_v_* value can easily lead to poor fluid flow in cartilage, but a high *P_v_* value can easily lead to cartilage brittleness, resulting in fractures, cartilage loss, and other diseases [10,11,12]. For health reasons, the *P_v_* values of cartilaginous materials should be as close as possible to that of natural cartilage, which must be between 30% and 47% [15,16,37,38].

**Figure 3:**
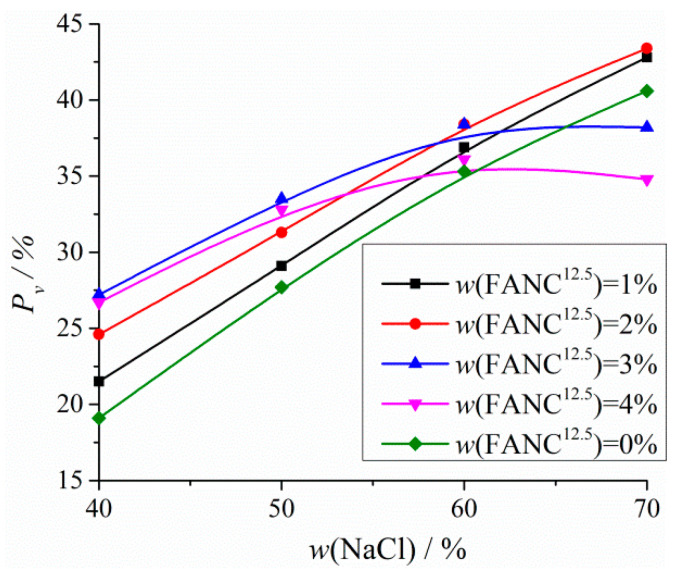
The *P_v_* values of porous UHMWPE/FANC^12.5^ specimens.

The porosity (*P_v_*) values of porous UHMWPE/FANC^12.5^ specimens with varying weight contents of FANC^12.5^ as the addition of NaCl particles increased from 40% to 70% are depicted in Figure 3.

The *P_v_* values of porous UHMWPE/FANC^12.5^ specimens clearly increased as the addition of NaCl particles increased from 40% to 70%, when the mass percentage of FANC^12.5^ was 0% to 2%. More pores occurred in porous UHMWPE/FANC^12.5^ specimens due to there being more NaCl particles that can be filtered from specimens with the increase in NaCl particles. *P_v_* reached a maximal value as the addition of NaCl particles approached an optimal value of 60%, and the mass percentage of FANC^12.5^ was between 3% and 4%. It is worth noting that smaller additions of FANC^12.5^ are more favorable for pore formation than a higher addition of FANC^12.5^. In fact, FANC^12.5^ easily agglomerates and becomes tangled as FANC^12.5^ is added at a higher level (i.e., >3%), which may cause NaCl particles to be wrapped up; thus, more NaCl particles cannot be filtered out by distilled water, resulting in a decrease in porosity [37,38]. The *P_v_* of the porous UHMWPE/FANC^12.5^ specimens were all above 30% when the addition of NaCl particles exceeded 60% and could reach 38.4% as FANC^12.5^ and NaCl particles were added at 3% and 60%, respectively.

Table 1 summarizes the tensile strength (*δ_f_*) values of porous UHMWPE/FANC^12.5^ specimens with varying weight contents of FANC^12.5^ and NaCl particles. As expected, *δ_f_* values of the porous UHMWPE/FANC^12.5^ specimens decreased gradually with the same contents of FANC^12.5^ as the addition of NaCl particles increased from 40% to 70%. The decrease was attributed to the increase in porosity caused by the increased addition of NaCl particles [39,40]. Tensile strengths of UHMWPE, UHMWPE with 40% NaCl, and UHMWPE/FANC^12.5^ with 1% FANC^12.5^ and 40% NaCl were 21.02 MPa, 17.1 MPa and 15.2 MPa, respectively. It is shown that tensile strength of UHMWPE/FANC^12.5^ depends on the additional amount of FANC^12.5^ and the porosity of UHMWPE/FANC^12.5^ [16,17,18,19,20,21]. When the additional amount of FANC^12.5^ was relatively low, e.g., 1%, the decrease in tensile strength of UMWPE/FANC^12.5^ was mainly due to the decrease in the orientation degree of the UHMWPE molecular chain caused by FANC^12.5^. At the same time, the increase in tensile strength of UHMWPE/FANC^12.5^ caused by the rigidity of FANC^12.5^ molecular chain can be ignored due to the small amount of FANC^12.5^. When the added amount of FANC_12.5_ increased from 2% to 4%, the tensile strength of UHMWPE/FANC^12.5^ increased from 16.3 MPa to 18.2 MPa. The reason for this is that the increase in tensile strength of UHMWPE/FANC^12.5^ caused by the rigidity of the molecular chain of FANC^12.5^ exceeded the decrease caused by the decrease in the orientation degree of the UHMWPE molecular chain. Therefore, at the same porosity, the tensile strength of UHMWPE/FANC^12.5^ decreased first and then increased with the gradual increase in the content of FANC^12.5^.

Considering the important effect of porosity on properties, porous UHMWPE/FANC^12.5^ specimens prepared with 3% FANC^12.5^ and 60% NaCl were determined to be more suitable for cartilaginous materials for subsequent research.

**Figure 4:**
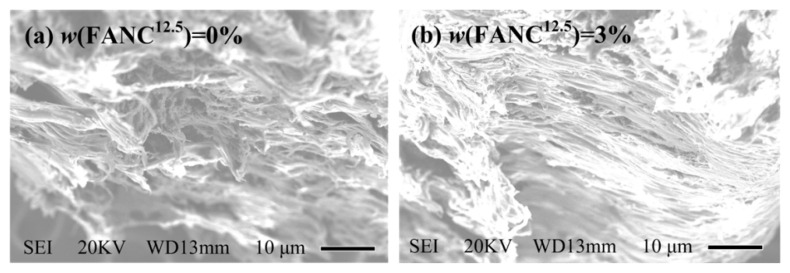
SEM images of the fractured surface of porous UHMWPE/FANC^12.5^ specimens with the addition of NaCl particles at 60%. The magnification is 3000×. (**a**) UHMWPE with no FANC^12.5^, (**b**) UHMWPE/FANC^12.5^ with the addition of FANC^12.5^ at 3%.

SEM images of the fractured surface of porous UHMWPE/FANC^12.5^ specimens with the 60% addition of NaCl particles and 0% and 3% weight contents of FANC^12.5^ are depicted in Figure 4. A few short, discontinuous cracks are clearly visible on the fractured surface of UHMWPE specimens prepared without FANC^12.5^ and the 60% addition of NaCl particles (see Figure 4a). This phenomenon demonstrated that the above specimens will be fragmented into blocks under fracture processes, as long as the fracture energy is very small. In contrast, there are many long, continuous, parallel cracks which are evenly distributed on the fractured surface of UHMWPE specimens prepared with the 60% addition of NaCl particles and a 3% weight content of FANC^12.5^ (see Figure 4b). This means that a larger fracture energy must be overcome during the fracture process; *δ_f_* values of porous UHMWPE/FANC^12.5^ specimens prepared with the 60% addition of NaCl particles and the 3% weight content of FANC^12.5^ were greater than those of the other specimens.

### 3.3. Surface Morphology and Wettability

**Figure 5:**
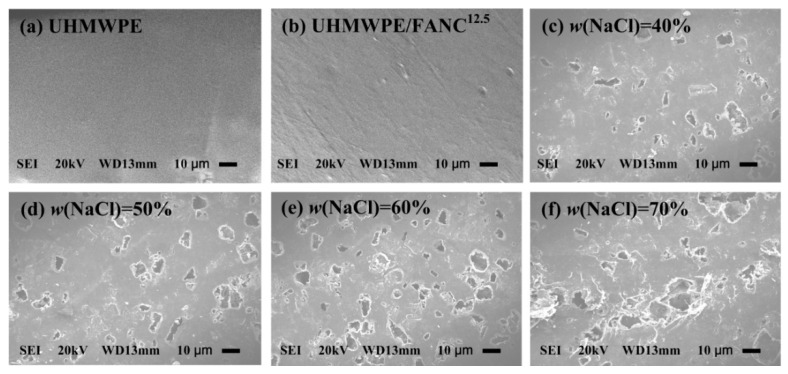
SEM of the surface of UHMWPE, UHMWPE/FANC^12.5^, and porous UHMWPE/FANC^12.5^ specimens with 3% weight contents of FANC^12.5^. The magnification is 1500×. (**a**) UHMWPE with no any additives, (**b**) UHMWPE/FANC^12.5^ with no NaCl, (**c**) UHMWPE/FANC^12.5^ (FANC^12.5^ 3%, NaCl 40%), (**d**) UHMWPE/FANC^12.5^ (FANC^12.5^ 3%, NaCl 50%), (**e**) UHMWPE/FANC^12.5^ (FANC^12.5^ 3%, NaCl 60%), (**f**) UHMWPE/FANC^12.5^ (FANC^12.5^ 3%, NaCl 70%).

SEM images of the surface of UHMWPE, UHMWPE/FANC^12.5^, and porous UHMWPE/FANC^12.5^ specimens with varying weight contents of FANC^12.5^ as the addition of NaCl particles increased from 40% to 70% are depicted in Figure 5.

The smooth and regular surface of pure UHMWPE is attributed to its long, non-polar -CH_2_- molecular chain. The surface of UHMWPE/FANC^12.5^ does not change obviously compared with that of pure UHMWPE, although there are more active groups, such as -OH, -COOH, on the surface of UHMWPE/FANC^12.5^ after adding PE-g-MAH. There are a large number of pores with different sizes distributed irregularly on the surface of porous UHMWPE/FANC^12.5^ specimens. It is worth noting that the *P_v_* of the surface of porous UHMWPE/FANC^12.5^ specimens (see Figure 5c–f) is greater than that of the original UHMWPE not filled with an additive agent (see Figure 5a). In the meantime, the numbers and pore sizes of the pores of the surface of porous UHMWPE/FANC^12.5^ specimens increased with the addition of NaCl particles and increased from 40% to 60% (see Figure 5c–e). However, the sizes of the pores of the surface of porous UHMWPE/FANC^12.5^ specimens kept increasing, whereas the numbers of the pores did not increase significantly, or sometimes even decreased with the increasing addition of NaCl particles from 60% to 70% (see Figure 5e,f). As evidenced by porosity analyses in the previous section, FANC^12.5^ is easily agglomerated and becomes tangled as FANC^12.5^ is added at higher levels, which can cause NaCl particles to be wrapped up so that more NaCl particles cannot be effectively filtered out.

**Figure 6:**
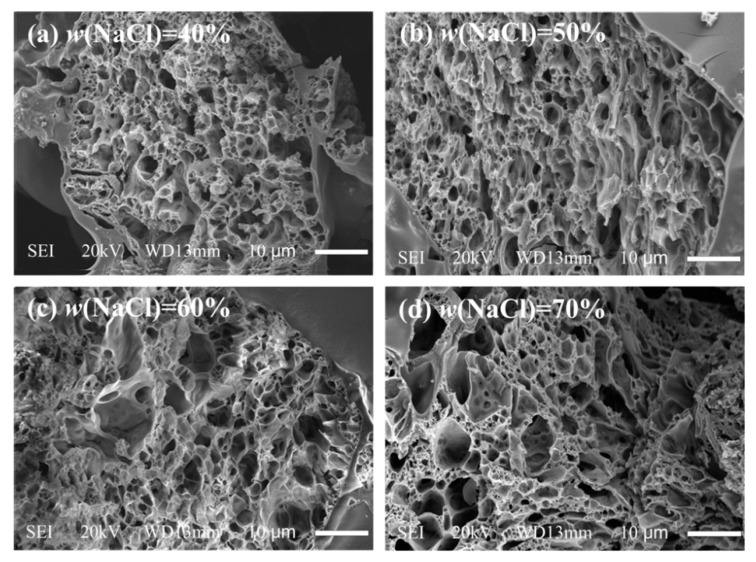
SEM images of the porous texture of porous UHMWPE/FANC^12.5^ specimens with a 3% weight content of FANC^12.5^. The magnification is 3000×. (**a**) UHMWPE/FANC^12.5^ (FANC^12.5^ 3%, NaCl 40%), (**b**) UHMWPE/FANC^12.5^ (FANC^12.5^ 3%, NaCl 50%), (**c**) UHMWPE/FANC^12.5^ (FANC^12.5^ 3%, NaCl 60%), (**d**) UHMWPE/FANC^12.5^ (FANC^12.5^ 3%, NaCl 70%).

SEM images of the porous texture of UHMWPE/FANC^12.5^ specimens with a 3% weight content of FANC^12.5^ are depicted in Figure 6. Typical irregular pore features with dimensions of 30–120 μm in diameter and interconnections between holes were observed in the brittle fractured surface of porous UHMWPE/FANC^12.5^ (see Figure 6a–d). Moreover, some nested meshes can be observed in the interior of the microholes. The porous structure mentioned above is of great benefit to porous UHMWPE/FANC^12.5^. For example, these pores are conducive to the growth of new tissue from the surface to the inside of the material, promote the flow of blood and improve the hydrophobicity of the material. Furthermore, when the amount of NaCl particles is more than 60%, the shape and diameter of pores are still irregular, but the proportion of pores with large pore size increases significantly (see Figure 6c,d), which leads to defects due to insufficient hardness and increased brittleness.

**Figure 7:**
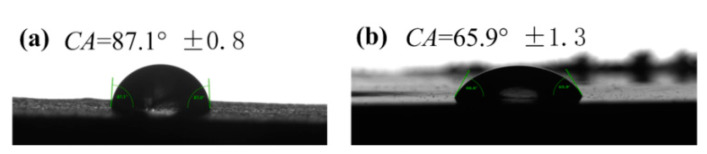
Static water contact angle (CA) of water droplets on the different specimen surfaces: (**a**) on UHMWPE; (**b**) on porous UHMWPE/FANC^12.5^. The immersion time was 4 s for these samples.

Static water contact angles (CAs) and photographs of water droplets on the different specimen surfaces are depicted in Figure 7.

Pure UHMWPE is hydrophobic due to its non-polar carbon chain, exhibiting a contact angle of 87.1°. For the porous UHMWPE/FANC^12.5^ prepared by incorporating 1% FANC^12.5^ and 40% NaCl, the contact angle decreased sharply to approximately 65.9°. Wenzel’s wetting equation is as follows:

(2) $$\cos\theta_{w}=r\cos\theta_{y}$$

where *θw* and *θy* are the apparent and intrinsic contact angle, respectively, and r is the ratio of the real surface area to the projected surface area and definitely greater than 1. Considering this information and the monotonous decrease in cosθ against θ from 0° to 180°, we can say that the rough surface structure makes the hydrophilic (*θy* < 90°) surface even more hydrophilic (*θw* < *θy*). Therefore, the hydrophilicity of porous UHMWPE/FANC^12.5^ was improved by hydrophilic groups on the surface of FANC^12.5^, such as -OH, C=O [41].

### 3.4. Biocompatibility

**Figure 8:**
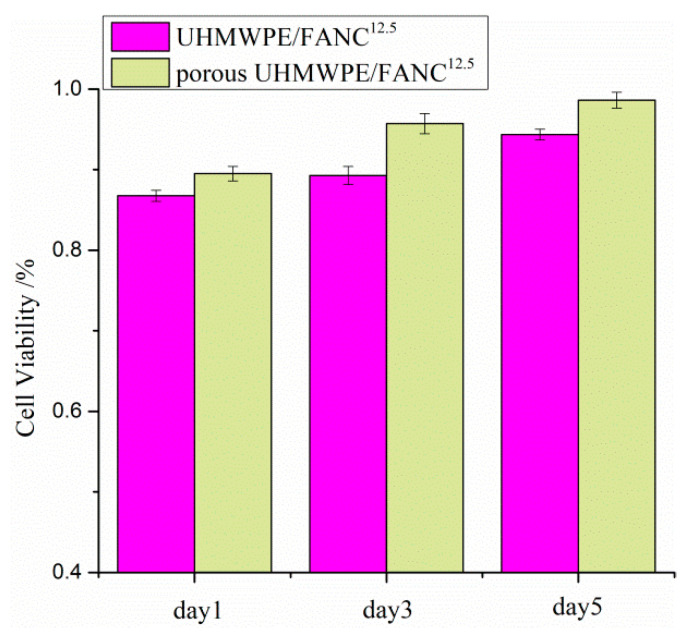
Cell viability of NIH 3T3 cells incubated with UHMWPE/FANC^12.5^ and porous UHMWPE/FANC^12.5^ (*p* < 0.05).

Porous UHMWPE/FANC^12.5^ specimens with appropriate mechanical strength are expected to be used as replacement cartilage material in biomedical applications. Therefore, cytotoxicity of the porous UHMWPE/FANC^12.5^ specimens is an essential criterion to assess before offering them as cartilage replacement materials. Using the proliferation of 3T3 cells on the surface of porous UHMWPE/FANC^12.5^ specimens, the cytotoxicity was studied by MTT assays. Viability values of the studied cells on porous UHMWPE/FANC^12.5^ specimens are shown in Figure 8. It can clearly be observed from the cell viability measurements that the number of viable cells gradually increased over time, exhibiting better biocompatibility of porous UHMWPE/FANC^12.5^ specimens. Interestingly, viability of the cells on the porous UHMWPE/FANC^12.5^ was greater than that of porous UHMWPE without modification by FANC^12.5^, clearly indicating that porous UHMWPE/FANC^12.5^ is more naturally biocompatible than porous UHMWPE without modification by FANC^12.5^.

**Figure 9:**
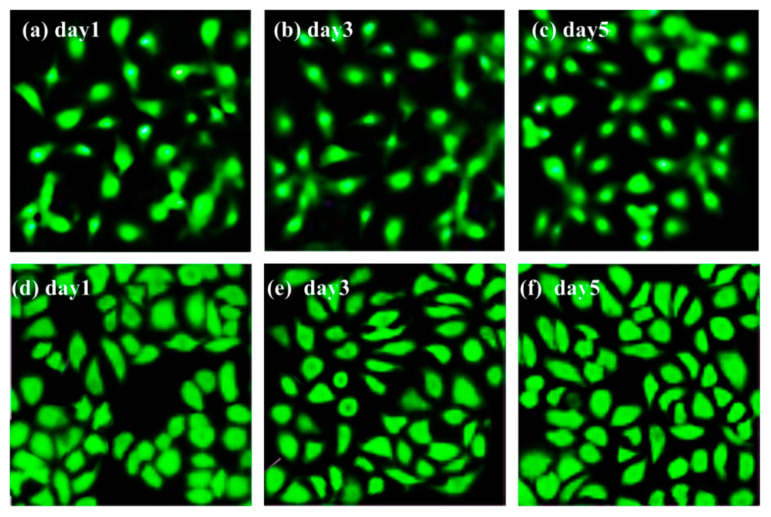
Fluorescence images of proliferated cells on different specimens: (**a**–**c**): porous UHMWPE; (**d**–**f**): porous UHMWPE/FANC^12.5^.

Fluorescence images of proliferated cells on porous UHMWPE/FANC^12.5^ and porous UHMWPE specimens are shown in Figure 9. Clearly, the proliferated cell density on porous UHMWPE/FANC^12.5^ and porous UHMWPE gradually became greater with prolonged incubation times (see Figure 9a–f). In addition, the proliferated cell density on the porous UHMWPE/FANC^12.5^ is greater compared to that on porous UHMWPE without modification with FANC^12.5^ after the same incubation time. It can further be explained that the porous UHMWPE/FANC^12.5^ samples are more biocompatible than the porous UHMWPE without modification with FANC^12.5^. The morphology of pores was unique in the porous UHMWPE/FANC^12.5^, where uniform pores were available for cell growth. Better pore formation and hydrophilicity of porous UHMWPE/FANC^12.5^ are responsible for this location-specific cell growth, and greater cell growth in the porous UHMWPE/FANC^12.5^ is noticed, which can be seen from cell viability and cell imaging investigations.

## 4. Conclusions

ATANC was prepared using activated nanocarbons (ANCs) etched with H_2_SO_4_/HNO_3_ (1:3 *v*/*v*). FANC*^x^* was prepared by grafting PE-g-MAH onto the ATANC surface. The optimal mass ratio (i.e., *x*) of PE-g-MAH to ATANC was determined to be 12.5:1 through FT-IR and SEM analysis. A novel, porous UHMWPE composition was successfully prepared by incorporating suitable contents of FANC*^x^* and pore-forming agent during the hot-pressing process of medical UHMWPE powder. The tensile properties of the porous UHMWPE were significantly improved due to the appropriate addition of FANC^12.5^. A large number of pores with different sizes were distributed irregularly on the surface and inside the porous UHMWPE/FANC^12.5^ specimens. The best tensile strength and porosity value for the porous UHMWPE/FANC^12.5^ specimens with a 3% content of FANC^12.5^ and the 60% addition of NaCl particles reached 14.3 MPa and 38.4%, respectively. The prepared porous UHMWPE/FANC^12.5^ exhibited excellent hydrophilicity, with a CA of 65.9°. The excellent biocompatibility of porous UHMWPE/FANC^12.5^ is due to its porous structure and hydrophilicity, caused by activated nanocarbon. Porous UHMWPE/FANC^12.5^ specimens with sufficient mechanical strength and hydrophilicity are promising materials to be used as cartilage replacement material for biomedical applications.

## Figures and Tables

**Table 1:** Tensile strength values (*δ_f_*) of porous UHMWPE/FANC^12.5.^

Content of FANC^12.5^ in UHMWPE/FANC^12.5^ (wt.%)	*δ_f_* (MPa)			
	*w* (NaCl) = 40%	*w* (NaCl) = 50%	*w* (NaCl) = 60%	*w* (NaCl) = 70%
0	17.1 ± 0.12	15.3 ± 0.14	10.2 ± 0.19	5.6 ± 0.24
1	15.2 ± 0.16	13.4 ± 0.16	8.5 ± 0.21	3.8 ± 0.26
2	16.3 ± 0.14	14.3 ± 0.16	9.3 ± 0.21	4.9 ± 0.24
3	17.6 ± 0.09	15.7 ± 0.13	14.3 ± 0.15	6.2 ± 0.21
4	18.2 ± 0.08	16.9 ± 0.11	15.1 ± 0.14	11.1 ± 0.15

Note: *δ_f_* of UHMWPE without NaCl addition is 21.02 ± 0.07 MPa.
